# Prediction of aboveground grassland biomass on the Loess Plateau, China, using a random forest algorithm

**DOI:** 10.1038/s41598-017-07197-6

**Published:** 2017-07-31

**Authors:** Yinyin Wang, Gaolin Wu, Lei Deng, Zhuangsheng Tang, Kaibo Wang, Wenyi Sun, Zhouping Shangguan

**Affiliations:** 1 State Key Laboratory of Soil Erosion and Dryland Farming on the Loess Plateau, Institute of Soil and Water Conservation, Chinese Academy of Sciences and Ministry of Water Resources, 712100 Yangling, Shaanxi P.R. China; 2 University of Chinese Academy of Sciences, Beijing, 100049 P.R. China; 3 State Key Laboratory of Soil Erosion and Dryland Farming on the Loess Plateau, Northwest A&F University, Yangling, Shaanxi 712100 P.R. China; 4 State Key Lab. of Loess and Quaternary Geology, Institute of Earth Environment, Chinese Academy of Sciences, Xi’an, Shaanxi 710075 P.R. China

## Abstract

Grasslands are an important component of terrestrial ecosystems that play a crucial role in the carbon cycle and climate change. In this study, we collected aboveground biomass (AGB) data from 223 grassland quadrats distributed across the Loess Plateau from 2011 to 2013 and predicted the spatial distribution of the grassland AGB at a 100-m resolution from both meteorological station and remote sensing data (TM and MODIS) using a Random Forest (RF) algorithm. The results showed that the predicted grassland AGB on the Loess Plateau decreased from east to west. Vegetation indexes were positively correlated with grassland AGB, and the normalized difference vegetation index (NDVI) acquired from TM data was the most important predictive factor. Tussock and shrub tussock had the highest AGB, and desert steppe had the lowest. Rainfall higher than 400 m might have benefitted the grassland AGB. Compared with those obtained for the bagging, mboost and the support vector machine (SVM) models, higher values for the mean Pearson coefficient (R) and the symmetric index of agreement (λ) were obtained for the RF model, indicating that this RF model could reasonably estimate the grassland AGB (65.01%) on the Loess Plateau.

## Introduction

Grasslands are indispensable terrestrial ecosystems^1–4^ for maintaining the ecological balance of arid and semi-arid regions under global climate change^5–7^. Increases in land-use intensity along with the uncertain risks from extreme climate events^8, 9^ have disturbed the native grassland successional processes. The Loess Plateau is an ecologically vulnerable area in China that is experiencing one of the most rapid rates of soil erosion in the world^10, 11^. Soil and water conservation and ecological restoration projects on the Loess Plateau have been widely valued by all sectors of society; in particularly, the “Grain for Green” project has been implemented by the Chinese government since 1999 to restore vegetation on steep, previously farmed lands and convert them to forests and grasslands^12, 13^. As a result of vegetation restoration on the Loess Plateau, sediment discharge into the Yellow River had declined to approximately 0.2 billion tons by 2013^14^, but researchers have found that revegetation was threatening the sustainability of water resources^15^. Assessment of the plant aboveground biomass (AGB) on the Loess Plateau is necessary to achieve sustainable vegetation restoration.

Remote sensing (RS) technology is a popular tool for estimating grassland AGB due to its ability to rapidly and continuously collect data over large areas^16–19^. Barrachina *et al*.^20^ employed Landsat TM-5 data to estimate the AGB in mountain meadows and pastures, and Li *et al*.^21^ developed a pure vegetation index model to predict the grassland AGB in the Inner Mongolian region of China. These studies indicated that AGB assessment using RS data is feasible, but the study areas were so different from the Loess Plateau that the fit of these models in that context cannot be validated. Newly launched satellites, such as Landsat 8, can potentially be used for quantifying ecosystem biomass^22^. For example, Dong *et al*.^23^ utilized Landsat 8 data to assess winter wheat biomass, and Dube *et al*.^24^ applied Landsat 8 images to estimate forest biomass. However, these authors focused on small-scale biomass estimation, and the existing Landsat 8 images of sufficient quality in 2013 could not cover the entire area of the Loess Plateau. Therefore, we utilized Landsat 5 images to acquire the vegetation index values used in this study.

A random forest (RF) model^25, 26^, which is a combination of multiple decision trees, is one example of a machine learning algorithm. Idowu *et al*.^27^ found that a machine learning algorithm might be more effective than a linear regression model for multi-variable models. Thus, it might be possible to effectively predict grassland AGB by combining an RF model with RS data. Previous researchers have measured the grassland AGB based on field experiments^28, 29^ but have not assessed the grassland AGB of the entire Loess Plateau because grasslands are widely distributed in this region^30^.

We attempted to predict the grassland AGB across the Loess Plateau, to understand the large-scale spatial characteristics of grasslands in this region by addressing the following questions. (1) Can an RF model be used to predict the grassland AGB on the Loess Plateau using meteorological and RS data? (2) What is the spatial distribution of the grassland AGB on the Loess Plateau? (3) How does the grassland AGB vary along the rainfall gradient? (4) How well does an RF model perform based on an accuracy assessment?

## Results

### Spatial distribution of the grassland AGB on the Loess Plateau

**Figure 1:**
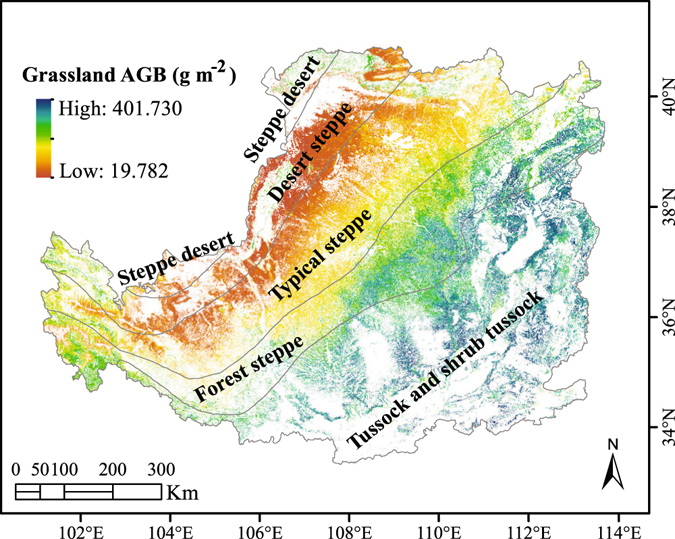
Spatial variation in the grassland AGB on the Loess Plateau. The map was generated using ArcMap Version 10.0 (http://www.esri.com/) and R Version 3.1.3 (https://www.r-project.org/).

Figure 1 shows the spatial distribution of the predicted grassland AGB on the Loess Plateau. The predicted grassland AGB decreased from east to the west across the plateau and ranged from 19.782 g m^−2^ to 401.73 g m^−2^, and it varied less in the longitudinal direction than in the latitudinal direction. The tussock and shrub tussock vegetation types had the highest AGB, followed by forest steppe; in general, the AGB in forest steppe is higher than the AGB in typical steppe, which is higher than the AGB in desert steppe. Both desert steppe and steppe desert had a relatively small AGB compared with the other vegetation types, and the lowest AGB was observed in the desert steppe.

### Rainfall affected the grassland AGB on the Loess Plateau

We divided the annual average rainfall from 2011 to 2013 (RM) into four types: arid (0–200 mm); semiarid (200–300 mm); alternate drying – wetting (300–400 mm) and semi-humid (>400 mm). Considering that rainfall might affect the grassland AGB in different ways under different percentages of bare area, we also categorized bare land into five types based on the percentage of the total area: B1 (<20%), B2 (20–40%), B3 (40–60%), B4 (60–80%), and B5 (>80%).

**Figure 2:**
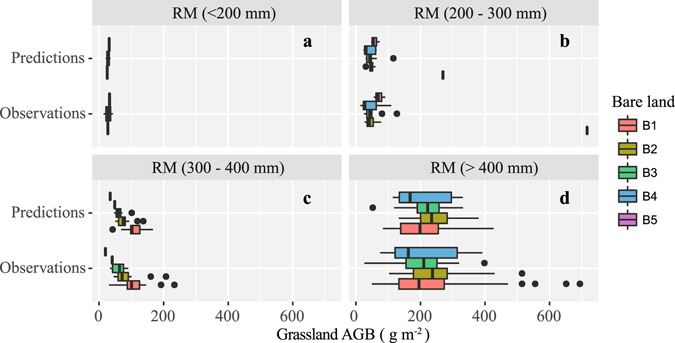
Observed and predicted grassland AGB in several of rainfall gradients (RM was annual average rainfall of 2011, 2012 and 2013) and bare land percentages (The total = 100%. B1: <20%; B2: 20–40%; B3: 40–60%; B4: 60–80%; B5: >80%).

The observed and predicted grassland AGB varied with the rainfall gradient (Fig. 2), and highest values for both of these variables were obtained when the rainfall was greater than 400 mm (Fig. 2d). If the rainfall was lower than 400 mm (Fig. 2a–c), the grassland AGB showed only slight changes along the rainfall gradient.

When the rainfall was in the range of 300 to 400 mm (Fig. 2c), the grassland AGB exhibited obvious patterns in response to different percentages of bare land, i.e., the lower the percentage of bare land, the higher the grassland AGB (both observed and predicted). However, this pattern was not apparent under other rainfall conditions (Fig. 2a,b and d).

### RF model validation

To validate the accuracy of the grassland AGB predicted by the RF model, we used Pearson’s coefficient (R) and the symmetric index of agreement (λ)^51^ to assess the correlation and agreement among the predicted and observed grassland AGB values. We also employed the mean error (ME), mean average error (MAE) and root mean square error (RMSE) to quantify the deviations among the predictions and observations. The observed grassland AGB (considered as 100%), which was randomly sampled as the validation set (from 10%~90%, at the intervals of 10%), and the training set was the remainder of the total set minus the validation set. Other machine learning models, such as bagging, mboost, and support vector machine (SVM), were also compared with the RF model.

**Figure 3:**
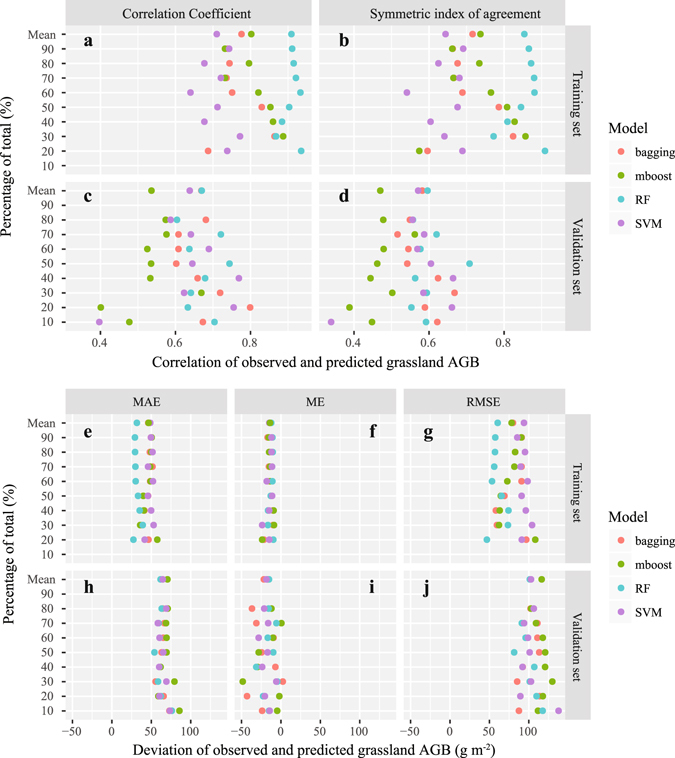
RF model validation and comparison with other machine learning models.

In the training set (Fig. 3a and b), the RF model had the highest mean R and λ, followed the mboost model, the bagging model, and the SVM model, which had the lowest value. In the validation set (Fig. 3c and d), the RF model had the highest mean R and λ, which were higher than those of the bagging model, and the bagging model values were higher than the SVM model values, which were higher than the mboost model values.

In the training set (Fig. 3e and g), the RF model had the lowest mean MAE and RMSE, and the same finding was obtained for the validation set (Fig. 3h and j). However, the mean ME of the four models (RF, bagging, mboost, and SVM) showed slight differences. Furthermore, the differences in the MAE, ME and RMSE of these four models were less easily distinguishable than the differences in the R and λ values.

### Partial dependence of various factors on grassland AGB

The factors used in the RF model made different contributions to the grassland AGB on the Loess Plateau, and their partial dependencies reflected their relationship to the grassland AGB. The predictive factors can be grouped into five categories: the normalized difference vegetation index (NDVI), the leaf area index (LAI), the fraction of photosynthetically active radiation (FPAR), rainfall and geographical location (longitude: x).

**Figure 4:**
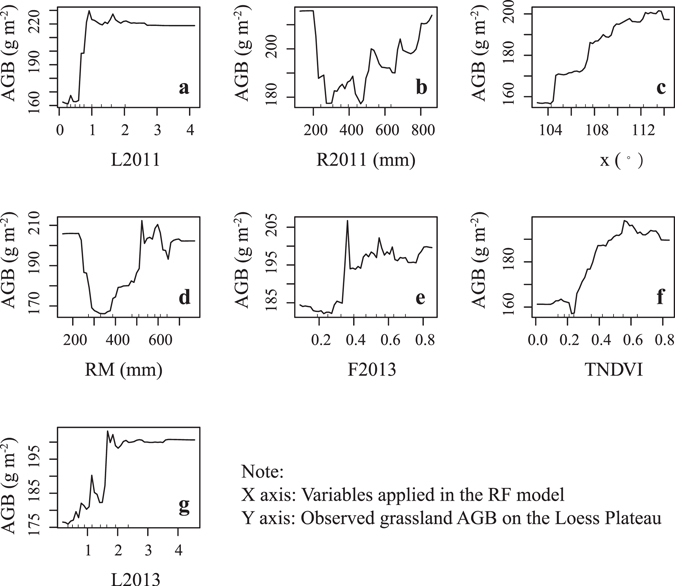
Partial dependence of various factors on observed grassland AGB. The meaning of factors referred to Table 2.

When the grassland AGB fell within the range of 160 to 220 g/m^2^, the NDVI and LAI were positively correlated with the grassland AGB (Fig. 4a,f and g). Ullah *et al*.^31^ found that the grassland AGB was positively correlated with the NDVI (R^2^ = 0.51), and Liang *et al*.^32^ concluded that an NDVI-based AGB model would be the most appropriate in their case study of the Three-River Headwaters Region in China. The above-mentioned results indicate that NDVI is an important factor in predicting the grassland AGB despite the low vegetation greenness of arid lands.

Previous researchers have used LAI and FPAR for crop modelling^33, 34^; thus, we used these variables in the grassland AGB model in this study. When the grassland AGB was higher than 180 g/m^2^, it was positively correlated with the FPAR (Fig. 4e), which showed that the FPAR could be a useful parameter in the estimation of AGB.

Grassland AGB was negatively correlated with rainfall when rainfall was lower than 400 mm and was positively correlated with rainfall when rainfall was higher than 400 mm (Fig. 4d). This result reveals that rainfall could be beneficial to grass growth in the semi-humid region of the Loess Plateau (rainfall >400 mm), but in the arid region (rainfall <200 mm), it might be difficult for grass to utilize rainfall. Extreme rainfall in the semi-arid region of the Loess Plateau (200 mm <rainfall <300 mm) might remove loose soil and hinder grass growth. Because rainfall is the only source of soil moisture on the Loess Plateau, soil moisture is closely related to rainfall gradients^35, 36^. Under wet conditions, the surface soil moisture is mainly controlled by rainfall, but under dry conditions, it is controlled by the plant water content and soil texture^37, 38^. This finding might explain why rainfall affected the grassland AGB of the arid/semiarid region and the semi-humid region of the Loess Plateau in different ways.

As shown in Fig. 4c, the grassland AGB increased with increasing longitude. At a large scale, the geographical location determines rainfall, and rainfall affects the soil water. The increase in soil water from the northwest to the southeast on the Loess Plateau^39^ could explain the spatial patterns of the grassland AGB.

### Image sources might affect the prediction accuracy

The spatial resolution of the predicted grassland AGB map in this study was 100 m, but the TM images (30 m), rainfall images (100 m) and MODIS images (500 m) had different spatial resolutions, which might result in error propagation. Over a large area, it is relatively difficult to collect all data at the same spatial resolution, and we usually tend to set the image resolution as high as possible. Considering the extensive computation requirements of this study, the spatial resolution was as high as could be expected, although it could be improved in future research.

As mentioned above, all of the images were collected on different dates in summer; thus, the grass conditions might have varied and could be another reason for the deviation of the predicted grassland AGB from the observational AGB. Data assimilation provided a way to integrate the RS images acquired from different satellites^40–42^; thus, the prediction accuracy might be improved if we consider this in the RF model.

### Comparison with other research

Jia *et al*.^43^ estimated the grassland biomass in northern China. Specifically, they collected field measurements and RS data during 2001-2005 and calculated the R^2^ values of the observations and predictions (R^2^: 0.54–0.66). The data collected in this study were more current (2011–2013). Liang *et al*.^32^ modelled the alpine grassland AGB and found that their multi-factor approach (latitude, longitude and grass cover) could reasonably estimate the AGB (63%); in this study, these factors explained 65.01% of the variation in the grassland AGB on the Loess Plateau. Næsset *et al*.^44^ estimated the forest biomass in a 365.6-km^2^ region based on Lidar data and attained R^2^ values in the range of 0.05 to 0.64 range, and Fayad *et al*.^45^ studied the forest AGB based on data from the optical geoscience laser altimeter system (GLAS) and found that R^2^ varied from 0.12 to 0.66. These results indicated that changes should be applied to the new RS platform and techniques for estimating the AGB of both forest and grassland, particularly the grassland AGB on the Loess Plateau because it is sensitive to environmental changes.

## Methods

### Study area

**Figure 5:**
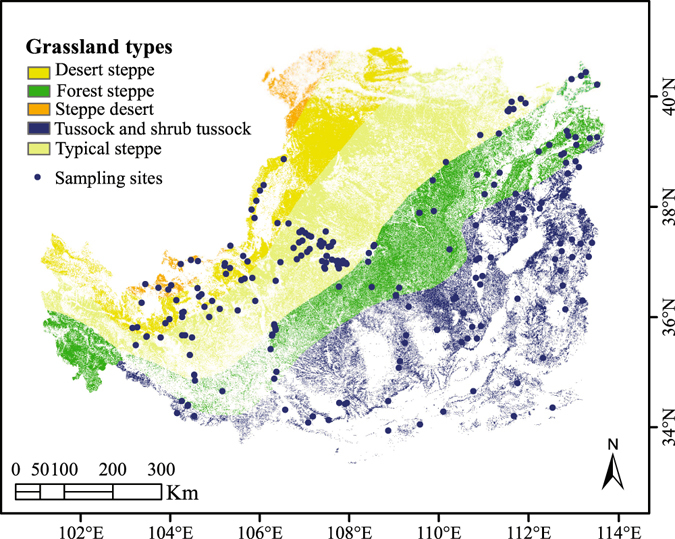
Sampling sites and grassland types on the Loess Plateau. The map was generated using ArcMap Version 10.0 (http://www.esri.com/).

The Loess Plateau covers an area of 0.64 million square km in central China and exhibits the typical characteristics of severe soil erosion and severe drought^46^. This region is dominated by a continental monsoon climate, with an average annual temperature ranging from 4.3 °C to 14.3 °C^47^ and a mean annual precipitation ranging from 200 mm to 750 mm^48^. In addition, extreme climate events have exacerbated the ecological imbalances in this region, but a reasonable increase in the AGB could mitigate environmental deterioration to a certain extent. The study area was the grassland on the Loess Plateau, which covers an area of 240,948 square km and accounts for nearly 1/3 of the total area of the plateau (Fig. 5).

### Collected data

The data used for the prediction of the grassland AGB on the Loess Plateau were a combination of observational data from a quadrat inventory and remote-sensing data, which can be summarized as follows:

1. Grassland inventory data A total of 233 grassland samples were collected across the Loess Plateau (Fig. 1) in summer from 2011 to 2013, and the study sites were located far from roads and villages to avoid human disturbance. At each sampling site, we assessed a 100-m line transect to identify a representative section and established 1 × 1-m quadrats at 20-m intervals. For each quadrat, the latitude, longitude, elevation, grass species, plant coverage and grass types were recorded. The aboveground parts of the green plants were then collected and dried at 65 °C for biomass determination by weight. The measured AGB ranged from 13.89 g m^−2^ to 716.17 g m^−2^, and the tested biomass data were split into two parts, one for training the RF model and the other for validating the predicted grassland AGB.
2. Thematic Mapper (TM) data (Table 1) Thematic Mapper data were acquired from the Landsat 5 satellite platform with a spatial resolution of 30 m (visible wavelengths), and images covering the Loess Plateau were downloaded from the United States Geological Survey (USGS) website (http://earthexplorer.usgs.gov/). All of the TM data were processed using the quick atmosphere correction within the Environment for Visualizing Images (ENVI 5.0) software package. The red band (wavelength: 620 nm~690 nm) and infrared band (wavelength: 760 nm~960 nm) were used to calculate the NDVI^49^ as follows:
  1 $${\rm{NDVI}}=({\rm{infrared}}\,{\rm{band}}-{\rm{red}}\,{\rm{band}})/({\rm{infrared}}\,{\rm{band}}+{\rm{red}}\,{\rm{band}})$$

**Table 1:** Acquisition dates and locations of TM and MODIS images.

Landsat 5 TM (Applied to calculate NDVI)						Terra MODIS (Applied to calculate FPAR and LAI)	
Date of image acquisition	WRS2 path	WRS2 row	Date of image acquisition	WRS2 path	WRS2 row	Date of image acquisition	Horizontal and vertical tile number
2011-08-18	124	32	2011-06-27	128	34	2011-07-04	h25v05h26v04h26v05h27v05
2010-08-15	124	33	2010-09-12	128	35	2011-07-12	
2010-08-15	124	34	2011-06-27	128	36	2011-07-20	
2011-06-15	124	35	2011-06-27	128	37	2011-07-28	
2011-06-15	124	36	2011-06-18	129	31	2011-08-05	
2011-06-15	124	37	2011-06-02	129	32	2011-08-13	
2010-07-05	125	32	2011-06-18	129	33	2011-08-21	
2010-09-23	125	33	2011-06-02	129	34	2011-08-29	
2011-08-09	125	34	2010-07-17	129	35	2012-06-01	
2011-07-08	125	35	2010-07-17	129	36	2012-06-09	
2011-07-08	125	36	2011-08-05	129	37	2012-06-17	
2011-07-08	125	37	2011-08-28	130	31	2012-06-25	
2011-09-01	126	31	2011-08-28	130	32	2012-07-03	
2011-06-13	126	32	2011-08-28	130	33	2012-07-11	
2010-07-12	126	33	2011-08-28	130	34	2012-07-19	
2010-07-12	126	34	2011-07-27	130	35	2012-07-27	
2011-07-15	126	35	2009-08-06	130	36	2012-08-04	
2011-07-15	126	36	2011-07-18	131	33	2012-08-12	
2011-09-01	126	37	2009-08-13	131	34	2012-08-20	
2011-06-04	127	31	2009-07-28	131	35	2012-08-28	
2011-07-22	127	32	2009-07-28	131	36	2013-08-05	
2011-08-07	127	33	2011-08-26	132	33	2013-08-13	
2011-08-07	127	34	2011-08-10	132	34	2013-08-21	
2010-06-17	127	35	2009-06-17	132	35	2013-08-29	
2011-06-04	127	36	2011-08-26	132	36		
2011-06-27	128	31	2011-08-01	133	33		
2011-07-13	128	32	2011-06-14	133	34		
2011-06-11	128	33	2011-06-14	133	35
3. MODIS-Terra, MOD15A2H FPAR and LAI data (Table 1) Moderate-resolution imaging spectroradiometer (MODIS) data (MOD15A2H version 6, MODIS Level 4) were acquired during the morning from the MODIS-Terra satellite. We downloaded eight-day composite products (500-m resolution) for 2011, 2012 and 2013 from the National Aeronautics and Space Administration (NASA) website (http://www.nasa.gov/). The downloaded data were used to extract the FPAR and LAI products using the MODIS Reprojection Tool (MRT).
4. Topographic data Digital elevation modelling (DEM) data with a 30-m horizontal spatial resolution from the ASTER GDEM version 2.0 product covering the Loess Plateau were downloaded from the USGS website (http://earthexplorer.usgs.gov/). We processed the data using the ArcGIS10.0 toolbox (Environmental Systems Research Institute, Inc., ESRI) to determine the slope of the Loess Plateau.
5. Climate data Quality-controlled climate data collected during 2011~2013 from 64 meteorological stations on the Loess Plateau were available from the National Climate Centre of the China Meteorological Administration (http://www.nmic.gov.cn). The data included the average monthly temperature, average monthly precipitation, and annual maximum and annual minimum temperature. The meteorological station point data were interpolated to fitted surfaces (100-m pixel cells) over the Loess Plateau using the ANUSPILN package, which contains FORTRAN programmes to fit the surfaces of one or more independent variables^50^. The average summer temperature and average summer precipitation were calculated using the monthly average temperature and precipitation values for June, July and August.
6. Auxiliary data A total of 24,094,252 control points were generated in the grassland to set the spatial resolution of the predicted AGB map to 100 m. The longitude and latitude of the control points were considered auxiliary data in this study, and the geographic projections of all maps were WGS 1984.

### RF model prediction

#### Variable selection for the RF model

The RF model was run using R 3.1.3 (http://www.r-project.org/) software^26^, and two parameters were involved in the optimization process: mtry and ntree. The parameter mtry represents the number of splits per node in each tree during the building process, and ntree is the number of decision trees or the number of bootstrap samples. The default mtry value is set to 1/3 of the number of independent variables^26^. In this study, the original data were log transformed to achieve normalization prior to model building.

**Figure 6:**
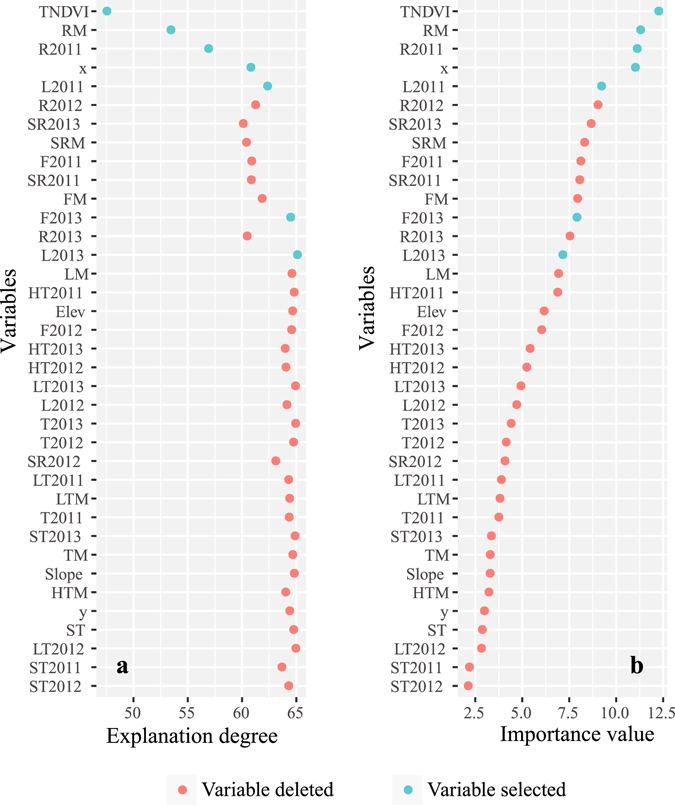
Variable selection for the RF model. The meaning of factors referred to Table 2.

A total of 38 primary variables were used at the beginning of the model-building process (Table 2). According to the importance value and the accumulated degrees of explanation of the variables calculated by the RF model, seven variables were finally selected using a stepwise method (Fig. 6); mtry was set as the default, and ntree was set to 300.

**Table 2:** Variable definitions in this study.

Factors	Definition	Description
Elev	Digital Elevation Model (DEM) on the Loess Plateau	Elevation (m)
Slope	Slope calculated by DEM on the Loess Plateau	Slope
TNDVI	NDVI calculated by TM data	Normalized difference vegetation index (NDVI)
L2011/L2012/L2013	Average LAI of 2011/2012/2013 summer	Leaf area index (LAI)
F2011/F2012/F2013	Average FPAR in 2011/2013/2013 summer	Fraction of Photosynthetically Active Radiation (FPAR)
FM	(F2011 + F2012 + F2013)/3	
R2011/R2012/R2013	Average rainfall of 2011, 2012,2013	Rainfall (mm)
RM	(R2011 + R2012 + R2013)/3	
SR2011/SR2012/SR2013	Average rainfall in 2011/2012/2013 summer	
SRM	(SR2011 + SR2012 + SR2013)/3	
HT2011HT2012/HT2013	Average of the high temperature in 2011,2012,2013	Temperature(°C)
HTM	(HT2011 + HT2012 + HT2013)/3	
LM	(L2011 + L2012 + L2013)/3	
LT2011/LT2012/LT2013	Average of the low temperature in 2011/2012/2013	
LTM	(LT2011 + LT2012 + LT2013)/3	
ST2011/ST2012/ST2013	Average temperature in 2011, 2012,2013 summer	
ST	(ST2011 + ST2012 + ST2013)/3	
T2011/T2012/T2013	Average temperature in 2011, 2012, 2013	
TM	(T2011 + T2012 + T2013)/3	
x	Longitude	Geographic location (°)
y	Latitude

#### Accuracy assessment

Error statistics were calculated for the predicted grassland AGB, and the residuals of the RF model were compared with the predictions obtained using other machine learning models (bagging, mboost, and SVM). The error statistics included the ME, MAE and RMSE, and their formulas are as follows:

2 $${\rm{ME}}=\frac{1}{N}\sum _{i=1}^{N}(Y-X)$$

3 $${\rm{MAE}}=\frac{1}{N}\sum _{i=1}^{N}|Y-X|$$

4 $${\rm{RMSE}}=\sqrt{\frac{1}{N}\sum _{i=1}^{N}{(Y-X)}^{2}}$$

In addition, R and λ were used to measure the correlation and agreement^51^ between the predicted grassland AGB and the observed values. The formula for R is as follows:

5 $${\rm{R}}=\frac{{\sum }_{i\,=\,1\,}^{N}(X\,-\,\bar{X})\,(Y\,-\,\bar{Y})}{\sqrt{{\sum }_{i\,=\,1\,}^{N}{(X-\bar{X})}^{2}}\sqrt{{\sum }_{i\,=\,1\,}^{N}{(Y-\bar{Y})}^{2}}}$$

6 $${\rm{\lambda }}=\frac{2}{{\sigma }_{X}\,/\,{\sigma }_{Y}+{\sigma }_{Y}\,/\,{\sigma }_{X}\,+\,{(\overline{X}-\bar{Y})}^{2}\,/{\sigma }_{X}{\sigma }_{Y}}\cdot r$$

In the above formulas, Y is the predicted grassland AGB, and X is the observed grassland AGB. The original data were split into several percentages for validation (10%~90% at 10% intervals). The error statistics and R values of the RF model were calculated within each percentage for comparison with the bagging, mboost and SVM models.
